# Genome-wide identification and expression analyses of the pectate lyase (PL) gene family in *Fragaria vesca*

**DOI:** 10.1186/s12864-023-09533-9

**Published:** 2023-08-03

**Authors:** Xiaolong Huang, Guilian Sun, Zongmin Wu, Yu Jiang, Qiaohong Li, Yin Yi, Huiqing Yan

**Affiliations:** 1 School of Life Sciences, Guizhou Normal University, Guiyang, 550001 China; 2 Key Laboratory of Plant Physiology and Development Regulation, Guizhou Normal University, Guiyang, 550001 China; 3 Key Laboratory of National Forestry and Grassland Administration on Biodiversity Conservation in Karst Mountainous Areas of Southwestern China, Guizhou Normal University, Guiyang, 550001 China; 4 Kiwifruit Breeding and Utilization Key Laboratory of Sichuan Province, Sichuan Provincial Academy of Natural Resource Science, Chengdu, 610015 China

**Keywords:** Pectate lyase, *Fragaria vesca*, Expression pattern, Anther, Fruit ripening

## Abstract

**Background:**

Pectate lyase (PL, EC 4.2.2.2), as an endo-acting depolymerizing enzyme, cleaves α-1,4-glycosidic linkages in esterified pectin and involves a broad range of cell wall modifications. However, the knowledge concerning the genome-wide analysis of the *PL* gene family in *Fragaria vesca* has not been thoroughly elucidated.

**Results:**

In this study, sixteen *PLs* members in *F. vesca* were identified based on a genome-wide investigation. Substantial divergences existed among *FvePLs* in gene duplication, *cis*-acting elements, and tissue expression patterns. Four clusters were classified according to phylogenetic analysis. *FvePL6*, *8* and *13* in cluster II significantly contributed to the significant expansions during evolution by comparing orthologous *PL* genes from *Malus domestica*, *Solanum lycopersicum*, *Arabidopsis thaliana*, and *Fragaria*×*ananassa*. The *cis*-acting elements implicated in the abscisic acid signaling pathway were abundant in the regions of *FvePLs* promoters. The RNA-seq data and in situ hybridization revealed that *FvePL1*, *4*, and *7* exhibited maximum expression in fruits at twenty days after pollination, whereas *FvePL8* and *FvePL13* were preferentially and prominently expressed in mature anthers and pollens. Additionally, the co-expression networks displayed that *FvePLs* had tight correlations with transcription factors and genes implicated in plant development, abiotic/biotic stresses, ions/Ca^2+^, and hormones, suggesting the potential roles of *FvePLs* during strawberry development. Besides, histological observations suggested that *FvePL1*, *4* and *7* enhanced cell division and expansion of the cortex, thus negatively influencing fruit firmness. Finally, *FvePL1-*RNAi reduced leaf size, altered petal architectures, disrupted normal pollen development, and rendered partial male sterility.

**Conclusion:**

These results provide valuable information for characterizing the evolution, expansion, expression patterns and functional analysis, which help to understand the molecular mechanisms of the *FvePLs* in the development of strawberries.

**Supplementary Information:**

The online version contains supplementary material available at 10.1186/s12864-023-09533-9.

## Background

The cell wall contributes to integrity and rigid but flexible outer surrounding for plant development, such as cell division, differentiation, and multi-cellular organ patterning [1]. It also acts as a barrier against pathogens. The cell wall structure comprises a cellulose-hemicellulose network with cross-linked pectin. Generally, pectin is a class of polysaccharide polymer with a linear backbone of 4-linked α-D-galactosyluronic acid residues, and its typical forms consist of homogalacturonan (HG), rhamnogalacturonan-I (RG-I), and rhamnogalacturonan-II (RG-II) [2]. Pectate lyase (PL, EC 4.2.2.2) that degrades pectin belongs to an endo-acting depolymerizing enzyme. It randomly cleaves α-1,4-glycosidic linkages by β-elimination to produce unsaturated 4, 5-oligogalacturonates in the presence of calcium, resulting in the depolymerization of HG, RG-I and RG-II, and cell wall breakdown [3].

*PL*s usually exist as a large family and are crucial in regulating organ-specific development. In tomato (*Solanum lycopersicum*), *Late anther tomato 56* (*LAT56*) and *LAT59* were first found to promote pollen tube growth by pectin degradation [4]. Besides, *Arabidopsis thaliana PL-like1* (*AtPLL1*) induces xylem formation, while *AtPLL12* increases lateral root development by degradation of the pectin-rich middle lamella and is also required for normal stomal dynamics [5]. Interestingly, *AtPLL13* (*PMR6*) influenced leaf morphology by promoting cell elongation [6, 7]. *PL*s in different plants also play divergent roles. For example, *Populus trichocarpa PL1-18* reduced the thickening of secondary cell walls of poplars [8]; *Gossypium hirsutum PL48* promoted cotton fiber cell elongation by facilitating cell wall loosening [9]; *Oryza sativa PL3* and *PL4* enhanced rice panicle development and male sterility [10].

Importantly, *PLs* are implicated in ripening-related cell wall modeling to accelerate fruit ripening, such as *S. lycopersicum*, cultivated octoploid strawberry *Fragaria*×*ananassa*, mango (*Mangifera indica*), and banana (*Musa acuminata*) [2, 11]. Antisense *plC* of *F. ananassa* cv. Chandler significantly enhances fruit firmness and prolongs shelf-life without affecting soluble solids. Interestingly, inhibiting transcripts of *PLs* significantly reduce the fruit ripening process at the transition stage from white to red [12]. Likewise, the transcripts of *MaPL1* and *MaPL2* are strongly induced during postharvest fruit ripening of bananas [2]. Besides, *PLs* also act as virulence factors of plant pathogens [13]. *Erwinia carotovora* and *Bacillus licheniformis PLs* caused soft-rot diseases and accelerated the maceration of plant tissues to pathogenesis [14]. Similarly, *Verticillium dahliae PL1* contributed to virulence and induced plant defense responses, such as strong programmed cell death [15]. Moreover, *AtPLL13* was responsible for powdery mildew susceptibility in Arabidopsis [6]. By contrast, silencing of *SlPL9* reduced susceptibility to the grey mound in tomatoes [16].

*PLs* expressions are generally regulated by hormonal signals. For example, one or more *AtPLLs* transcripts are altered by hormonal treatments. The elicitor of defense response methyl jasmonate even induces alternative splicing of *AtPLL8* into two transcripts [1]. The previous study mentioned that the most dramatic effects on the expression of *PLs* were produced by abscisic acid (ABA), heat, and ultraviolet [1]. It is worth noting that ABA strongly influences the expression of most *AtPLLs* in Arabidopsis. Similarly, ABA increases the transcripts of *FaPL* (*F. ananassa* ‘Elsanta’) by transcriptional factor SHATTERPROOF-like MADS-box to control fruit ripening [17]. Additionally, *AtPLL13* was induced by sensing auxin, resulting in size augments by cell expansion [6]. Also, the studies have predicted that the regions of all *GhPLs* promoters contain at least one of the auxin-responsive *cis*-elements [9]. Moreover, the application of auxin or ethylene significantly enhanced the expressions of *MaPL1* and *MaPL2* to promote banana ripening [18]. *Rosa hybrida* AP2/Ethylene-responsive factors (RhERF1 and RhERF4) bound to the promoter of the pectin-metabolizing gene to delay petal abscission [19]. Overall, *PLs* likely regulate plant development by responding to hormonal signals.

The woodland strawberry *Fragaria vesca* has been developed as a model of the Rosacea family due to its short life cycle and small genome size. The amenable *Agrobacterium*-mediated transformation makes it an ideal species for exploring gene functions [20]. Three *FvePLs* (*PLA*, *PLB*, and *PLC*) are identified in *F. vesca* by PCR amplifications and highly expressed in developing fruits to accelerate fruit softening [21, 22]. Nevertheless, there are no available genome-wide analyses of the *PLs* family in *F. vesca* to date.

By mining large-scale genome data, sixteen *FvePLs* were identified, and their phylogenetic relationships, gene structures, conserved domains, and collinearity were predicted. The *cis*-acting elements suggested that *FvePLs* were responsive to organic development, phytohormones, and biotic stress. The co-expression networks were constructed to provide new insights into gene functions and relationships underlying the molecular processes of strawberries. The expression profiles and in situ hybridization indicates functional divergence of *FvePLs* in different organs. Overall, our identification and characterization of *FvePLs* will broaden the understanding of the roles of *PLs* in the Rosacea family.

## Materials and methods

### Identification and characterization of *PL *genes in woodland strawberry *F. vesca*

The protein sequences of *F. vesca* (v4.0.a2) were downloaded from the Genome Database for Rosaceae (GDR) website at www.rosaceae.org/species/fragaria/fragaria_vesca. The Hidden Markov Model (HMM) profile of the Pec_lyase_C family (Pfam00544) was retrieved from the Pfam (http://pfam.xfam.org/) and was performed to search for the target proteins by HMMER 3.0 (http://hmmer.janelia.org/). The threshold of E-value for the HMMER search was set at 1e-10 to determine the potential FvePL proteins. Subsequently, all non-redundant sequences were checked and confirmed using the Simple Modular Architecture Research Tool (SMART) web server (http://smart.embl-heidelberg.de/) regarding the conserved domain. The coding domain sequences (CDS) and nucleotide sequences of the *FvePLs* were isolated from the GDR database. The *PL* genes were identified from the Arabidopsis Information Resource (TAIR, https://www.arabidopsis.org/) and tomato genome databases (http://solgenomics.net/), respectively.

The physicochemical properties of FvePL proteins were predicted using ExPASy Proteomics Server (http://web.expasy.org/protparam/), including molecular weight (MW), amino acid counts, isoelectric point (pI) values, grand average of hydropathicity (GRAVY) and subcellular localization. The conserved motifs of FvePL were detected using the motif analysis tool Multiple Em for Motif Elicitation (MEME, http://meme.nbcr.net/meme3/meme.html) with the default parameters.

### Phylogenic and syntenic analysis of *PL *gene family

The multiple alignments of the full-length amino acid sequences of all FvePLs were determined using ClustalX 2.1. A phylogenetic tree was constructed using MEGA7.0 (http://www.megasoftware.net.) and the maximum likelihood (ML) method with 1000 bootstrap replicates. The genome sequences and annotations of *Malus domestica* (GDDH13V1.1) and *F. ananassa* (FL15.89-25) were downloaded from the GDR database. Multiple Collinearity Scan toolkit MCscanX (http://chibba.pgml.uga.edu/mcscan2/) was used to identify syntenic and collinear regions and duplicated regions between the *FvePLs* in Arabidopsis, tomato, apple, and *F. ananassa*. The linear maps of syntenic analysis were visualized using TBtools [23]. A schematic diagram of the putative whole-genome duplication (WGD) or segmental duplication was constructed and linked by lines.

### Analysis of *cis*-element, expression patterns, and co-expression network of *FvePLs*

To investigate the *cis*-acting elements in the promoters of *FvePL* genes, the 2 kb upstream nucleotide sequences of *FvePL* were downloaded from the GDR database and analyzed by the NEW PLACE (https://sogo.dna.affrc.go.jp/cgi-bin/sogo.cgi). The transcripts of *FvePL* genes in various tissues were identified, including seed, leaf, seedling, carpel, anther, pollen, style, ovule, embryo, ghost, pith, cortex, receptacle, and fruit at different stages [20]. The transcript per million (TPM) reads data were downloaded from an eFP browser http://mb3.towson.edu/efp/cgi-bin/efpWeb.cgi [24]. The heatmap of expression normalized by log_2_(TPM) transformation was viewed using tbtools software [23]. The Pearson correlation coefficient (PCC) of genes was calculated based on the combined 46 existing RNA-seq libraries [25]. The positively correlated genes with *FvePLs* (cutoff 0.9) were screened and correlation analysis was conducted.

### *In situ* hybridization

This study used Yellow Wonder 5AF7 (YW5AF7) seedlings, the 7th generation inbred lines of *F. vesca*. The plants were grown in a greenhouse (16 h/8 h light conditions at 22 °C, at a relative humidity of 65%) [26]. For cytological observation, the petal, stem, leaf, anther, filament, and fruit at 15 days after pollination (DAP), 20 DAP, and 25 DAP were sampled, then fixed in RNase-free FAA solution (4% formaldehyde, 50% ethanol, and 10% acetic acid). The fixed tissues were dehydrated in ethanol series and embedded in paraffin wax. After dewaxing, rehydration, sealing, and staining, the tissues were observed and recorded. Cross-section slicing (8 μm) was performed by Leica RM2255 (Leica Inc., Buffalo Grove, IL, USA).

A gene-specific cDNA fragment of *FvePL1, 4, 7, 8*, or *13* was individually amplified using ISH-F/R primer for in situ hybridization (Additional file 1). Their PCR product was then cloned into the pGEM-T vector. A DIG RNA labelling Kit (Roche, German) was applied to the tissue paraffin Sect. [27]. Sense and antisense RNA probes were synthesized using SP6 and T7 RNA polymerase, respectively. In situ hybridization experiments were performed, including prehybridization, hybridization, washing, and detection [28]. Sides were photographed under a BX53 microscope (Olympus, Japan).

### Subcellular location

The amplified PCR products of *FvePL1, 4, 7, 8*, and *13* were cloned at the *Xba*І site of the pM999 vector to produce CaMV 35 S::*FvePLs*-GFP vectors. Protoplasts were isolated from the mesophyll of 4-week-old Columbia-0 (Col-0) ecotype Arabidopsis leaves [29]. For transfection, 200 µL protoplast was transferred into a 2 mL round-bottom microcentrifuge tube and mixed with 20 µL recombinant or control plasmid and 220 µL PEG solution [29]. After transfection, Arabidopsis protoplasts were incubated in the low light level at 22℃ for 16 h/8 h before examination by fluorescence microscopy. At the same time, the cell membrane was marked by chloromethyl-benzamidodialkyl carbocyanine (CM-Dil) at 10 µmol/L for 15 min. Images were acquired using a ZEISS LSM 710 fluorescence microscope (ZEISS, Germany).

### Vector construction and plant transformation

The full-length CDS of *FvePL1, 4*, or *7* was amplified using YW5AF7 leaves individually. The amplified PCR product was cloned at *Kpn*І and *Spe*І sites of the pMDC32 to generate the pMDC32-*FvePL-ox* constructs (Additional file 1). For RNAi, the partial coding sequences of *FvePL1, 4*, or *7* were cloned into pDS1301 to produce the pDS1301-*FvePLs*-RNAi construct. *Agrobacterium tumefaciens* GV3101 harboring pDS1301-*FvePL1*-RNAi construct was co-cultivated with cross-sectioned leaf slices from YW5AF7 plants. Strawberry transformation and regeneration were using previously published protocols [20, 30]. The stable transgenic lines were screened by 2 mg/L hygromycin and PCR verification. Pollen grains from newly opened flowers were collected and then stained with 3-(4,5-dimethylthiazol-2-yl)-2,5-diphenyl monotetrazolium bromide (MTT) solution to detect pollen viability [31]. Meanwhile, Agrobacterium GV3101 strains which harbored the overexpression or RNAi vector of *FvePL1, 4*, or *7*, respectively, were infiltrated into the YW5AF7 fruit flesh at the turning stage (20 DAP) using syringes according to the previous study [32]. At least thirty fruits of each genotype were injected.

### Measurement of fruit firmness

Fruit firmness was determined based on puncture strength and compression mass of fresh intact fruit using a TA.XTplusC Texture Analyzer (Stable Micro Systems, Surrey, UK). The system was equipped with texture profile analysis (TPA). Hardness was measured as the maximum penetration force (N) reached during tissue breakage. The maximum penetration force was set as 25 N [33]. The parameters used were as follows: pre-test speed 1.0 mm sec^–1^, test speed of 1.0 mm sec^–1,^ and post-test speed of 10.00 mm sec^–1^. The probe with 2 mm diameter was pressed into the intact fruit from the flesh to a depth of 5 mm. At least twenty fruits were selected for each genotype, and the average value was taken in a unit of N [34].

### Determination of pectin content

The content of pectin was determined using the carbazole colorimetric method [35]. The pectin was extracted by weighing 1.0 g leaf from *F. vesca* into a mortar, adding 1 mL 95% ethanol and thoroughly grinding to generate fine homogenate. Then, the mixture was added with 25 mL of 95% ethanol and boiled in a water bath for 30 min. After cooling to room temperature, the solution was centrifuged at 8000 rpm for 15 min and the precipitate was collected. Finally, 20 mL of distilled water was added and boiled at 50℃ for 30 min to dissolve the pectin. A 1.0 mL solution was mixed with 0.25 mL of 0.1% carbazole-ethanol solution, and 5.0 mL sulfuric acid was added within 6 s. The mixed solution was incubated at 85℃ for 20 min and cooled quickly, after which the absorbance at 525 nm was measured. The standard curves were plotted using different concentrations (0, 20, 40, 60, 80, 100 mg/L) of galacturonic acid standard solutions. The reference pectin contents measured by the sulfuric acid-carbazole colorimetry method were expressed as galacturonic acid equivalents. The water-soluble pectin was homogalacturonan and expressed as galacturonic acid equivalents, and total pectin was a sum of measured protopectin and water-soluble pectin. All experiments were performed in triplicate.

### qRT-PCR analysis

Total RNA extraction was performed by Mouhu et al. [36]. The cDNA for qRT-PCR was synthesized using an EasyScript® First-Strand cDNA Synthesis SuperMix (TransGen, China). We amplified PCR products in triplicate using PerfectStart® Green qPCR SuperMix (TransGen, China) in 10 µL reactions for qRT-PCR analysis. PCR was performed using the Bio-Rad CFX96 Touch real-time PCR Detection System (Bio-Rad, USA), and cycling conditions consisted of denaturation at 95℃ for 30 s, followed by 40 cycles at 95℃ for 5 s, annealing at 60℃ for 30 s, and extension at 72℃ for 30 s. We used *FvH4_4g24420* encoding glyceraldehyde-3-phosphate dehydrogenase (GAPDH) as an internal control. Specific primers for the *FvePLs* were listed in Additional file 1. The relative expression data were calculated using the 2^−ΔΔCt^ method [37].

### Statistical methods

Statistical analysis was done using SPSS v22.0 (IBM Corp., Armonk, NY, USA). The comparison between multiple samples was determined by One-way ANOVA using Tukey’s test, and significant differences at the *P* values < 0.05 level are indicated by different letters.

## Results

### Identification, physicochemical properties, and gene structures of *FvePLs*

To identify candidate *PLs*, an HMM search was performed against the *F. vesca* genome using a conserved Pec_lyase _C domain (Pfam00544). A total of 16 genes were identified as members of the *F. vesca PL* family and their integrities were further confirmed using the online CDD and SMART programs. The distributions of *FvePLs* were uneven across the six chromosomes of *F. vesca*. For example, the most abundant was Chr. 6 with five members, and no gene was set in Chr. 1. They were designated as *FvePL1* ~ *FvePL16* according to their chromosomal locations. Their physical and chemical properties showed that FvePLs ranged from 352 amino acids (aa) (FvePL9) to 504 aa (FvePL15) in length, corresponding to MW between 38.88 kDa (FvePL9) and 53.52 kDa (FvePL15) (Additional file 2). The theoretical *p*I varied from 5.96 (FvePL12) to 9.51 (FvePL3), in accordance with the optimum pH for cleaving reactions in vitro. Furthermore, fourteen PLs were predicted to have instability coefficients below 40, indicating that most FvePLs belong to stable proteins. Since the GRAVY index reflects the hydropathicity of the protein, the negative GRAVY values mean that all FvePLs are hydrophilic. The location of the FvePLs is predicted to be in cell walls, cell membranes, or chloroplasts.

**Fig. 1:**
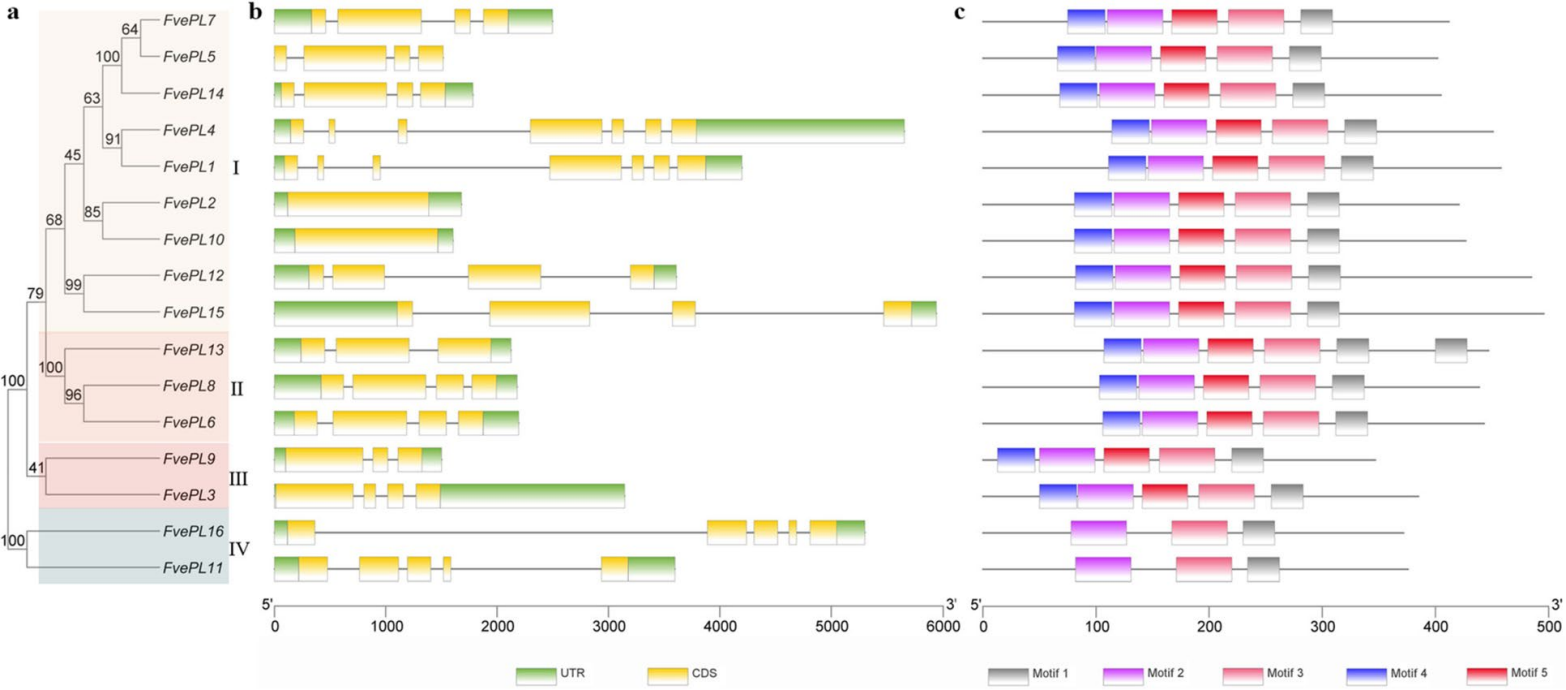
Phylogenetic relationships, gene structures and motif compositions of *FvePLs*. **a** The full-length FvePL sequences are aligned using ClustalW and the unrooted tree is generated using the MEGA7.0 by maximum likelihood (ML) method with 1000 bootstrap replicates. **b** Exon/intron structures of *FvePLs*. Green boxes, UTRs; yellow boxes, exon; black lines, introns. **c** Conserved motif analysis of FvePL by MEME. Different colored boxes represent different motifs

The phylogenetic distribution exhibited that *FvePLs* were divided into four major clusters (Fig. 1a). Cluster I and cluster II each contained nine (*FvePL1*, *2*, *4*, *5, 7, 10, 12, 14* and *15*) and three members (*FvePL6*, *8* and *13*). Additionally, two genes were categorized into Cluster III (*FvePL3* and *9*) or Cluster IV (*FvePL11* and *16*). To thoroughly uncover the structural traits of *FvePLs*, we constructed their intron-exon arrangements (Fig. 1b). The number of exons of the *FvePLs* ranged from one to seven. Briefly, most *FvePLs* in the same phylogenetic group displayed similar intron and exon lengths. However, a small proportion of *FvePLs* pairs in the same cluster exhibited different gene structures. For instance, *FvePL6* and *FvePL8* in cluster II both contained four exons and three introns. By contrast, *FvePL13* harbored three exons and two introns.

To further reveal the specific regions of FvePLs, the conserved motifs were predicted by MEME and the top five individual motifs were isolated (Fig. 1c). The length of motifs ranged from 29 to 50 aa (Additional file 3). FvePLs branched in the same phylogenetic cluster and contained similar motif compositions. For instance, all FvePLs in cluster I and II consisted of five motifs, suggesting that the resemblant functionality of these conserved motifs might participate in the homogenous biological processes of *F. vesca* development. It was worth noting that FvePL13 harbored two repeated Pec_lyase domains, implying an endo-acting solid depolymerizing capability.

### Synteny analysis of the *FvePLs *within different species

**Fig. 2:**
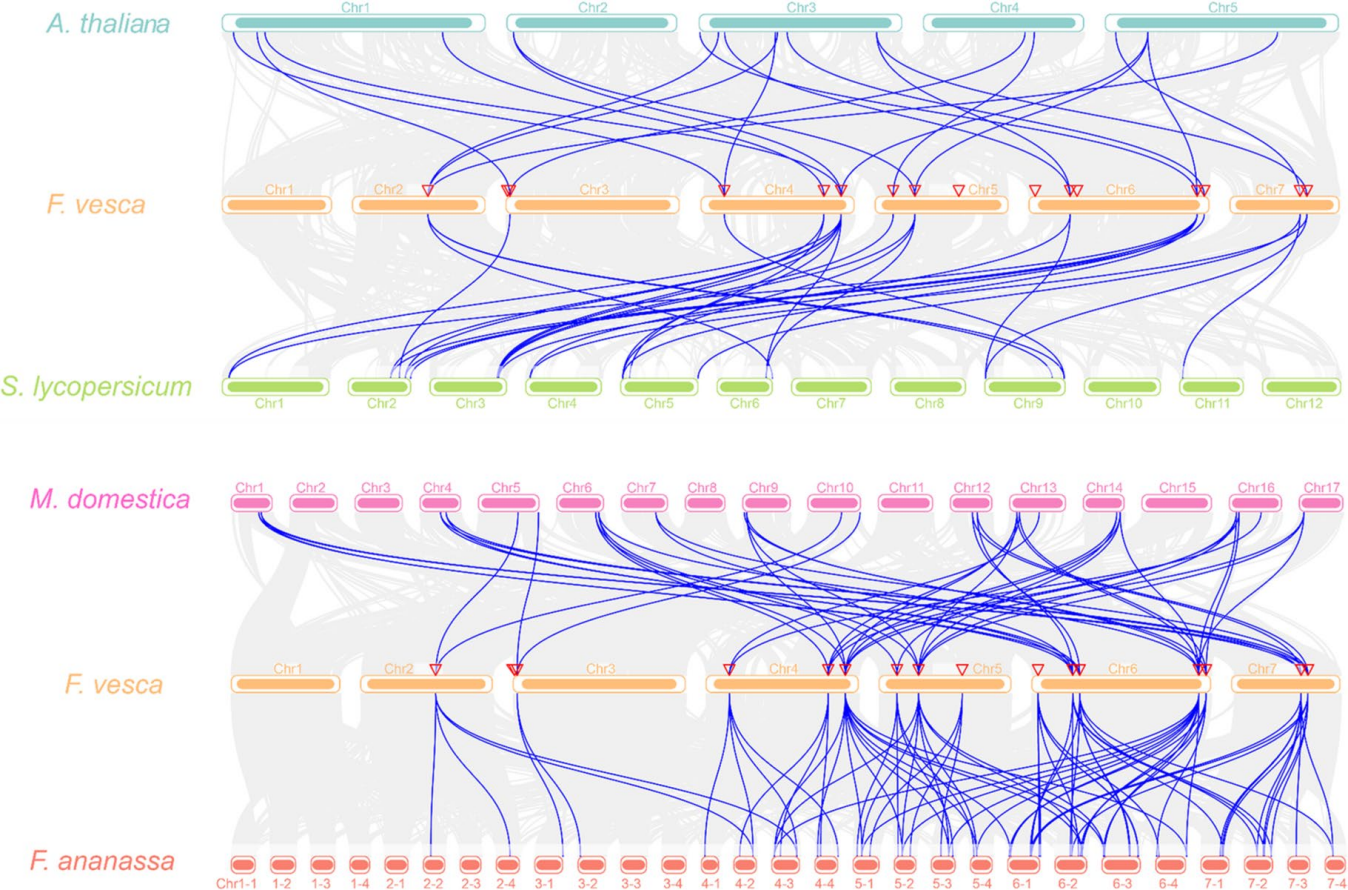
Synteny analyses between *PLs from F. vesca* and other species. *PLs* are anchored based on their positions on the chromosomes. Grey lines indicate collinear blocks between *F. vesca* and other plant genomes. In contrast, blue lines highlight syntenic *PLs* pairs between *F. vesca* and Arabidopsis, *S. lycopersicum*, *M. domestica* and *F. ananassa*

To understand the putative clues of evolutionary events, we surveyed the syntenic relationship among orthologous *PLs* from *F. vesca*, Arabidopsis, and *S. lycopersicum* (Fig. 2). The latter two species belong to well-characterized representative species. Orthologous genes of 12 *FvePLs* were found to be matched in both *Arabidopsis* and *S. lycopersicum*, except four genes (*FvePL2*, *9*, *10* and *12*), suggesting that most *FvePLs* might have existed before the ancestral divergence (Additional file 4). We individually detected 21 and 25 pairs of orthologous genes in Arabidopsis and *S. lycopersicum*. Each *FvePL* had only two or three orthologous genes in Arabidopsis. In contrast, some *FvePLs* had up to five orthologous genes in *S. lycopersicum*, implying that *FvePLs* were phylogenetically closer to *PLs* from *S. lycopersicum* than in Arabidopsis. Moreover, *FvePL6* and *FvePL13* were the most highlighted due to their high contribution to gene expression in evolution.

To further investigate the evolutionary relationships of *FvePLs* within the Rosaceae species, syntenic regions of the *PLs* genes in *Malus domestica* and cultivated octoploid strawberry *Fragaria ananassa* were performed. Many syntenic blocks were found based on one-to-more corresponding relationships of *FvePLs* with *PLs* from *M. domestica* and *F. ananassa*. Totally 47 pairs of orthologous genes were observed between *F. vesca* and *M. domestica*, including 13 genes (except *FvePL2*, *9* and *10*) and 24 *MdPLs*. Notably, more pairs of orthologous genes (80 pairs) were identified between *F. vesca* and *F. ananassa*, comprising 15 genes (except *FvePL2*) and 54 *FaPLs* (Additional file 4). The expansion of *PLs* in *M. domestica* and *F. ananassa* is mainly derived from segmental or whole-genome duplication (WGD). It is worth noting that *FvePL6*, 8 and *13* contributed the most significant expansions of *MdPLs* since each had six orthologous to *FvePLs*. Likewise, *FaPLs* even had ten copies orthologous to *FvePL6*, seven orthologous to *FveP8* and nine orthologous to *FveP13*, thus suggesting that *FvePL6*, *8* and *13* may be substantially crucial for the expansion of the *PL* gene family during evolution.

### *FvePLs *transcripts were responsive to specific organ development, hormonal, and biotic stresses

**Fig. 3:**
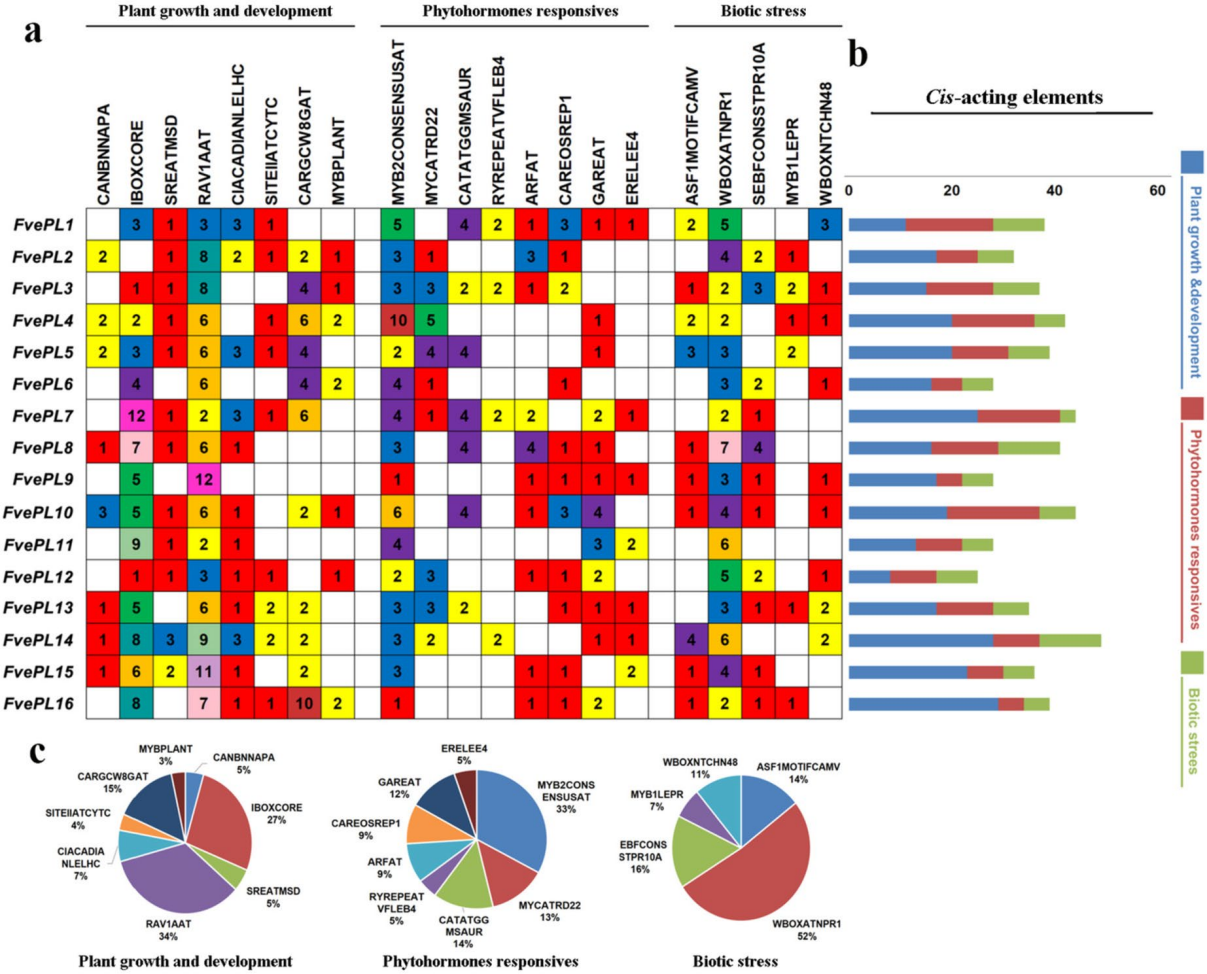
Identification of ***cis*** -acting regulatory elements in the promoter region of *FvePLs*. **a** The elements are divided into three main categories: plant growth development, phytohormone response and biotic stress. The numbers represent the frequency of the elements occurring in the promoter region. **b** The differently colored histograms represent the sum of *cis*-acting elements in each category. **c** Pie charts of different sizes indicate the proportion of each promoter element in each category

The *cis*-acting elements from 2 kb DNA sequences in the regions of *FvePLs* promoters were analyzed to gain insight into the potential functions and regulatory mechanisms of *FvePLs* during plant development (Additional file 5). Several growth and developmental elements were identified, including elements involved in anther and meristem specificity (SITEIIATCYTC), vascular tissue specificity (RAV1AAT), and phenylpropanoid synthesis (MYBPLANT) (Fig. 3a **and b**). Notably, RAV1AAT accounted for the most significant proportion (34%), followed by IBOXCORE (27%) and CARGCW8GAT (15%) (Fig. 3c). The high enrichment of RAV1AAT elements suggests that FvePLs may, in theory, be responsive to vascular tissue specificity and impact cell wall modification.

A variety of hormone-related elements were identified, including ABA-responsive (MYCATRD22 and MYB2CONSENSUSAT), auxin-responsive (CATATGGMSAUR), ethylene-responsive (ERELEE4), and GA-responsive (CAREOSREP1) elements, thus suggesting that various hormonal signals regulated *FvePL*s. In addition, the elements of MYB2CONSENSUSAT (33%) and MYCATRD22 (13%) involved in the ABA signalling pathway were the most abundant in the regions of the FvePLs promoters (Fig. 3c**)**.

Multiple biotic stress-related elements were observed, consisting of nonexpresser of PR genes 1 (NPR1), disease resistance protein (ASF1MOTIFCAMV), pathogen-induced enhancement of plant defense response (WBOXATNPR1), the regulator of expression of defense-related genes (MYB1LEPR) and the auxiliary response of defense genes (WBOXNTCHN48). Among the elements, WBOXATNPR1 had a great percentage (52%), suggesting that FvePLs could be responsive to defense responses (Fig. 3c).

### Spatial and temporal expression profiles of *FvePLs*

**Fig. 4:**
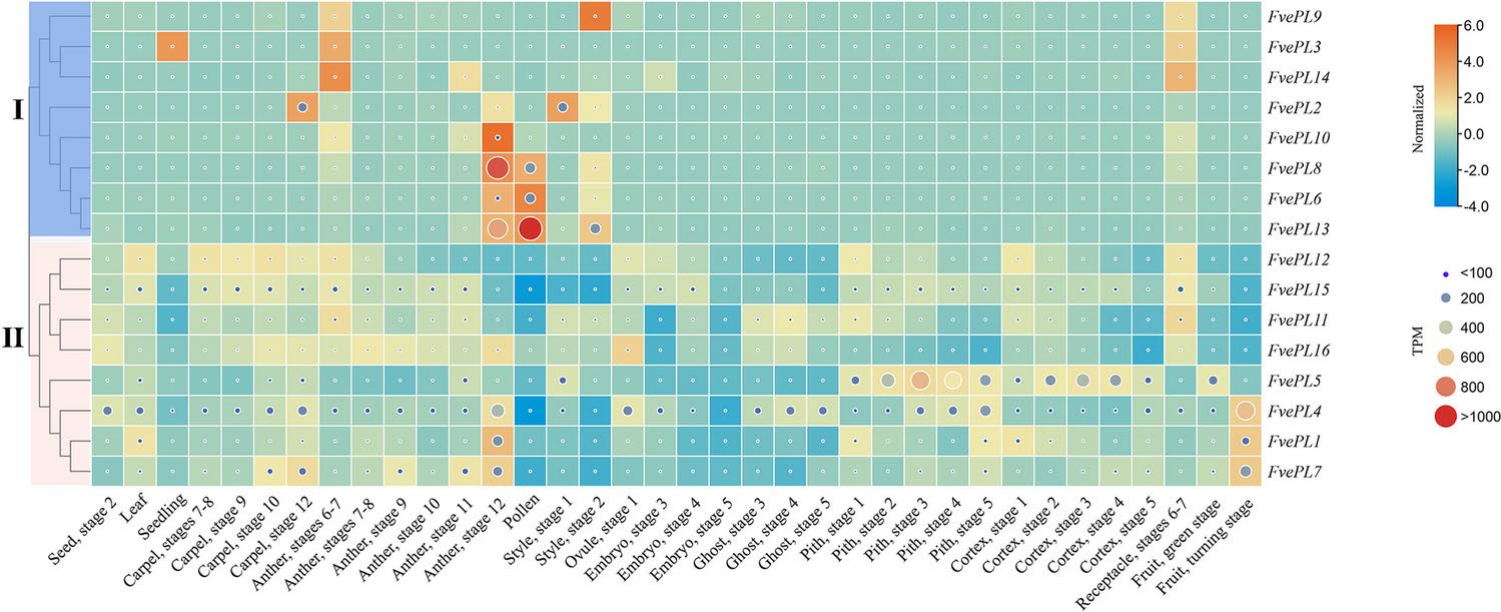
Spatial and temporal expression patterns of *FvePLs.* The data are normalized based on the mean expression value of each gene in all organs. The heatmap portrays the relative expressions after the log_2_ transformation indicated by the square. The orange, yellow, and blue squares indicate expressions in a high, medium, and low level, respectively. The circles reflect the TPM values and the larger circle indicates the higher expression value

A heatmap was drawn to visualize the expression profiles of individual *FvePL* based on previously published RNA-seq data. The divergent expression patterns of *FvePLs* were found in different organs, which were categorized into various stages based on structural and/or cytological events [26]. These results indicated a stringent developmental regulation of *FvePLs* expression (Additional file 6). The expression values of all *FvePLs* could be clustered into two groups. *FvePLs* in group I exhibited very low or no expression in most tissues). However, some of them were found to be preferentially expressed in specific tissues **(**Fig. 4**)**. For example, transcripts of *FvePL6* and *8* exhibited expressions only exclusively to anther at stage 12 when anthers are opaque yellow and appear fully differentiated. It should be noted that *FvePL13* was the most highlighted due to its maximum expression in pollens. The remaining eight constitutive expression *FvePLs* in group II displayed an extensive expression range. Several *FvePLs* in this group showed expressional activations in at least two or more tissues. The most conspicuous genes were *FvePL1*, *4*, and *7*, because of their maximum expression in fruits at the turning stage. Besides, *FvePL1* showed maximum expression in anther at stage 12 compared to other *FvePLs* in group II. It is also significantly expressed in the anther at stage 12 compared to other tissues, suggesting its critical role in pollen development. All data suggested that *FvePLs* played specialized roles in the development of strawberries.

### Co-expression network analysis of *FvePLs*

**Fig. 5:**
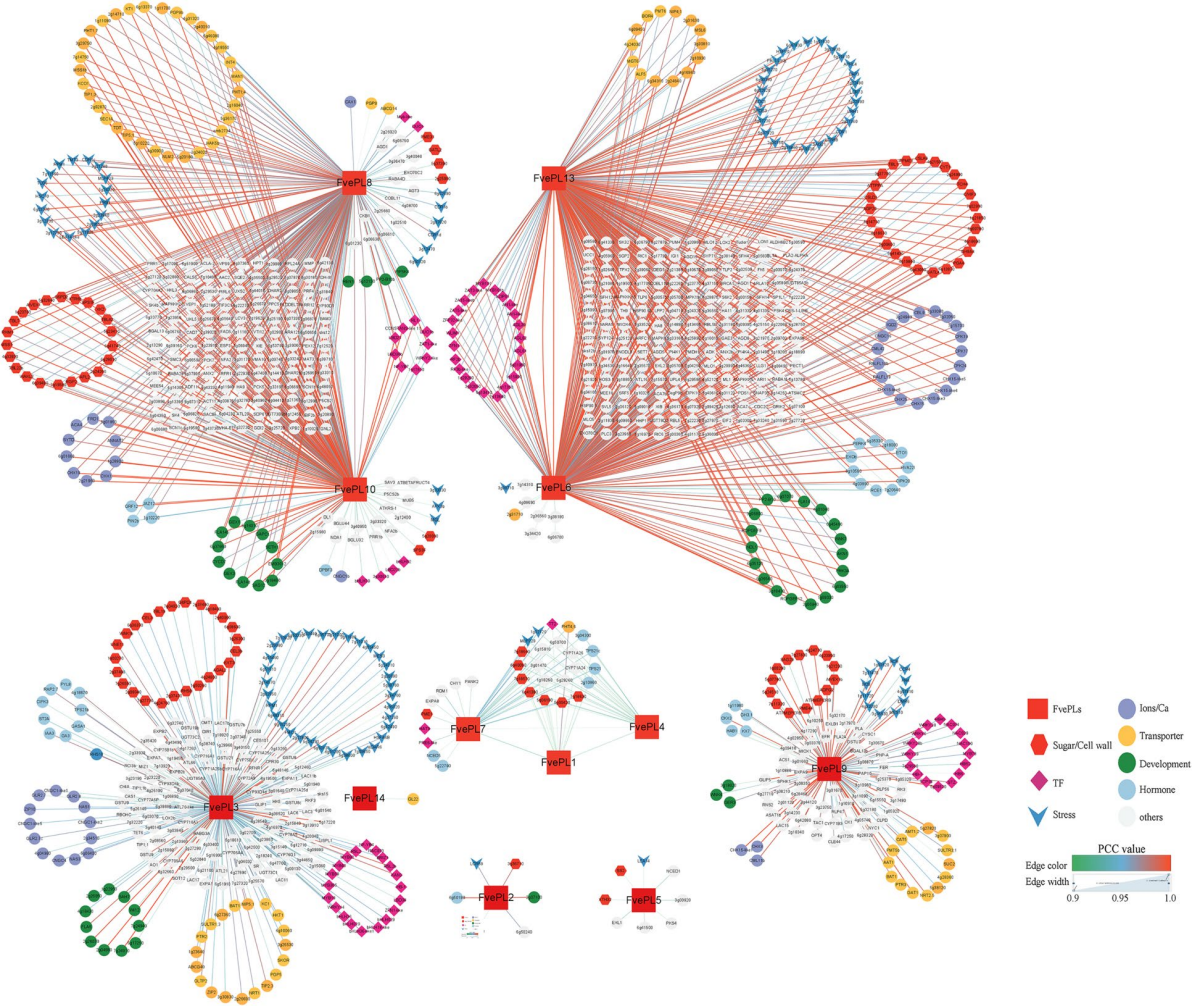
The co-expression network of *FvePLs* generated by RNA-seq data. A total of 1502 genes with a PCC higher than 0.9 are visualized by Cytoscape (v3.6.1). The darker red node color indicates higher PCC values. Different shapes represent corresponding annotations within the network

To explore and mine the possible roles of *FvePLs* in fruit development, we identified sets of neighborhoods of connected genes invoked by *FvePLs* that shared similar expression profiles. The co-expression networks were illuminated in accordance with the PCC (cutoff 0.9) and visualized using Cytoscape. Sixteen *FvePLs* correlated 3440 links with 2101 genes. Among these members, *FvePL6* has come out on top (727 genes), with *FvePL13* (707 genes), *FvePL8* (655 genes), *FvePL10* (618 genes), *FvePL3* (380 genes) following in second, third, fourth and fifth position respectively (Additional file 7). After removing unannotated genes, they were determined in eight clusters and correlated with 1502 genes. The functions of co-expressed genes were indicated in different colors and forms (Fig. 5). *FvePL8* and *10* were clustered into the same group. *FvePL6* and *13* were also gathered, and the group of *FvePL1, 4* and *7*, suggesting that they might share homogenous regulatory mechanisms or functions. Among the correlated genes, *FvePLs* exhibited more correlations with genes annotated as transcription factors (76 genes), transporters (71 genes), biotic/ abiotic stresses (65 genes), sugar/cell walls (63 genes), development (49 genes), ions/Ca^2+^ (43 genes), and hormones (33 genes). The data suggested that *FvePLs* played multiple and essential roles in strawberry development.

### FvePLs were localized in cell membranes or chloroplasts

**Fig. 6:**
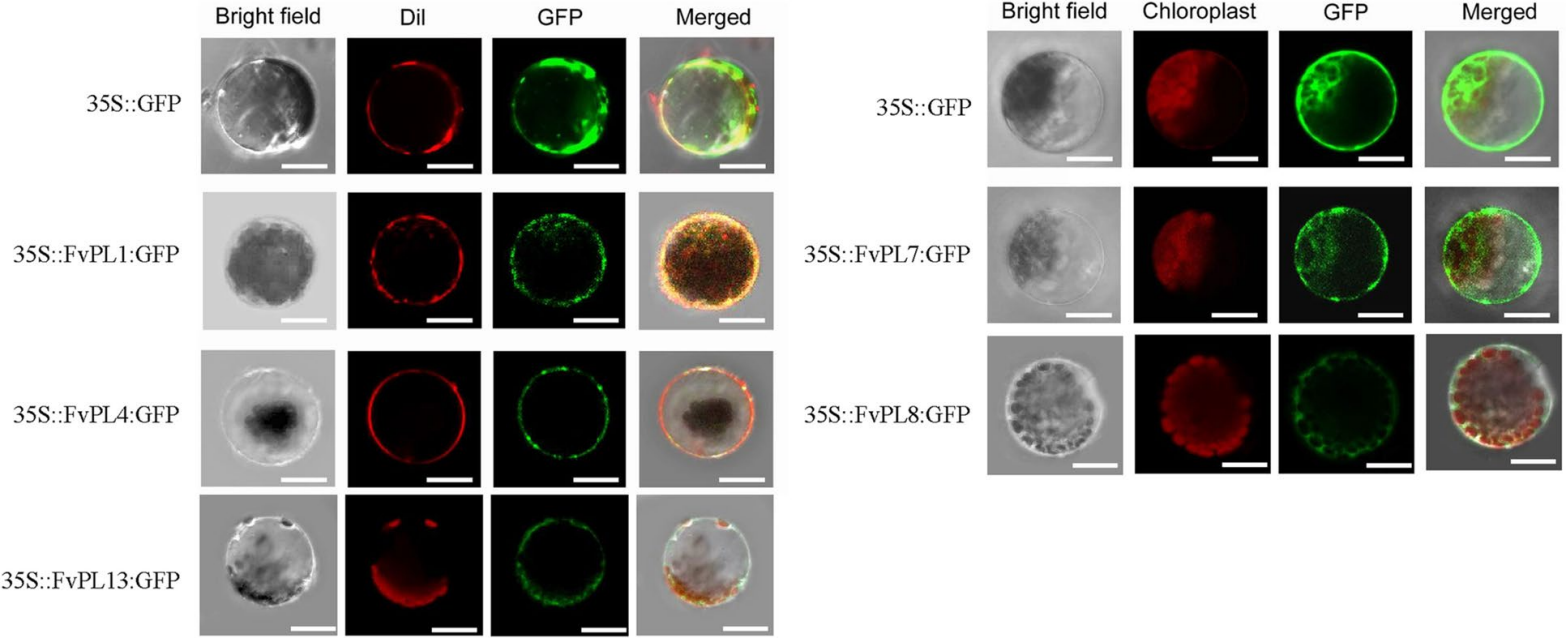
Subcellular localization of FvePL1, 4, 7, 8, and 13. Transient expression of 35S::GFP and 35S::FvePL1/4/7:GFP fusion protein in protoplasts. GFP alone (negative control) constructs were transiently expressed under the cauliflower mosaic virus 35S promoter, and the GFP signal was observed by confocal microscopy 16 h after transfection. The GFP fluorescence (green), chlorophyll autofluorescence (red), bright field, and the combined images are shown. Scale bar = 10 μm

To investigate the precise subcellular localization of the FvePLs, we selected five highly expressed *FvePLs* (*1*, *4*, *7*, *8*, and *13*). The vectors of 35 S::*FvePLs*:GFP plasmids were constructed, individually. Each was transformed into Arabidopsis protoplasts. Microscopic visualization showed that the GFP fluorescence signals of the control 35 S::GFP were presented in the whole cell, including the membrane and cytoplasm. In contrast, the CM-Dil staining of protoplasts was localized exclusively in the cell membrane. When transformed with 35 S::*FvePL1/4/13*:GFP, the strong green fluorescent signals were detected only in the cell membrane of protoplasts akin to the site as CM-Dil staining. However, the protoplasts expressing 35 S::*FvePL7/8*:GFP showed green fluorescent in the cell wall and a chloroplast localization similar to the red signals by chlorophyll autofluorescence (Fig. 6). These data revealed that FvePLs were localized in the cell membrane and/ or chloroplast.

### *FvePLs *exert diverse roles in anther, pollen, and fruit development

**Fig. 7:**
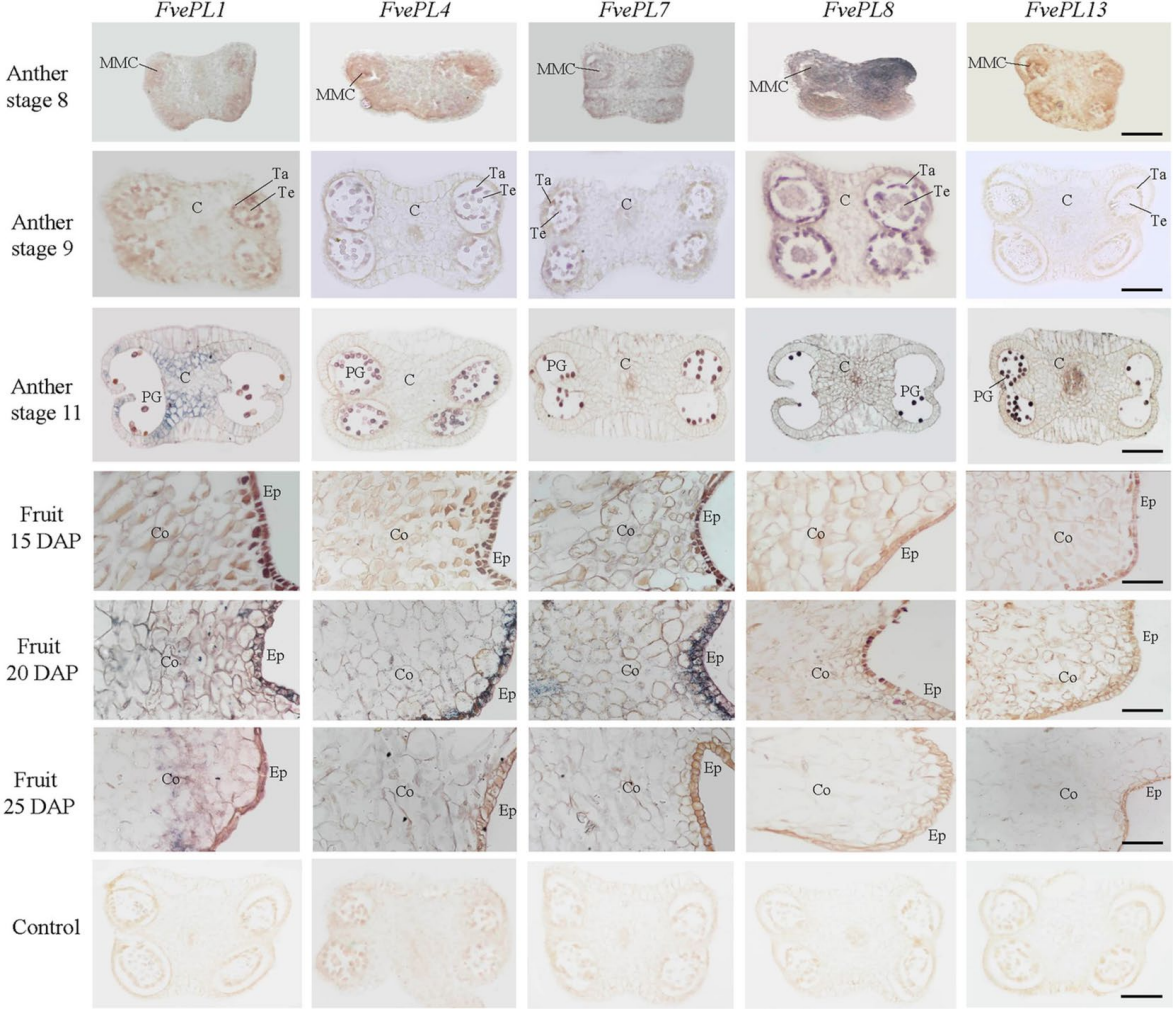
In situ hybridization analysis of *FvePL1, 4, 7, 8, and 13*. mRNA in situ hybridization of *FvePL1*, *4*, *7*, *8*, and *13* in anther at stage 8 (microspore mother cells appear), stage 9 (microspore mother cells enter meiosis, but resulting tetrads are tightly confined in locules), and stage 11 (mature anther), fruits at 15 DAP, 20 DAP and 25 DAP, as well as a control using the sense RNA probe expressed in the anther, DAP: days after pollination, Scale bar = 50 μm. PG, pollen grains; Co, cortex; Ep, epidermal cell; C, connective tissue

The same highly expressed five genes (*FvePL1*, *4*, *7*, *8*, and *13*) were made for further detailed investigations. *FvePL1*, *4*, *7*, *8*, and *13* expressions in anthers and fruits at different stages were determined by in situ hybridization (Fig. 7). The strong signals of *FvePL8* were found in the parenchymal cells of the anther, followed by *FvePL7* at anther stage 8 when microspores mother cells appeared and four locules are distinct. No obvious signals of *FvePL1, 4* and *13* were detected at the same stage, consistent with the RNA-seq data. Subsequently, microspore mother cells enter meiosis at stage 9, resulting in tetrads confined in locules and the middle layer degenerating. At this stage, *FvePL8* was preferentially expressed in endothecium, middle layer, and tapetum arising from the anther’s parietal and epidermal cells, suggesting its potential roles in degenerating of middle layers. The detected signals were also found in the tapetum and tetrads of *FvePL4* and *7*, thus facilitating the degradation of the primary cell wall in pollen mother cells (PMC) at the meiosis stage. Finally, the septum was degraded, and round tricellular pollen was formed at anther stage 11. The significantly visible signals of *FvePL1*, *4, 8* and *13* were displayed in pollen grains at this stage, suggesting their potential roles in the promotion of pollen penetration. We also detected obvious signals of *FvePL1* in the connected tissues of anthers and epidermis, implying that it may protect the anther or provide structural support.

**Fig. 8:**
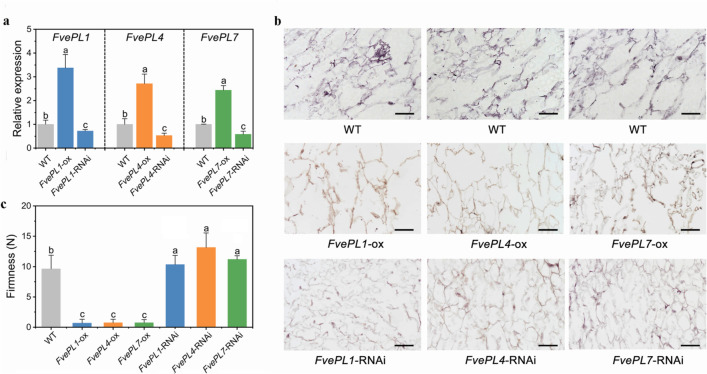
*FvePL1, 4 and 7* involved in fruit softening. a qRT-PCR analysis of *FvePL1*, *4* and *7* in wild-type, overexpression, and RNAi fruits at three days after injection. **b** Anatomical observation of wild type, *FvePL1/4/7*-overexpression, and -RNAi fruits at seven days after injection. **c** Fruit firmness at seven days after injection. Values represent the mean of three biological replicates. Scale bar = 50 μm

Despite of the high expression in pollen, there were no detected signals of *FvePL8* and *13* in fruits (Fig. 7). On the other hand, distinct hybrid signals of *FvePL1*, *4* and *7* were observed in the epidermal cell layer and parenchyma of the cortex cells at 20 DPA, corresponding to the higher expression in ripening fruits at the stage of turning from white to red [20]. The results represent the developmental regulation of the *FvePL* expressions. To explore *FvePLs* in fruit development, RNAi, and overexpression of *FvePL1*, *4* and *7* were obtained using agro-infiltrated into ‘Yellow Wonder’ fruits. The transcripts of *FvePL1*, *4* and *7* in overexpression lines were 3.42-, 2.86- and 2.67-fold increase of WT, respectively, whereas in *FvePL1/4/7-*RNAi were remarkably declined (Fig. 8a). Anatomical observations were performed to investigate the structural basis of the fruit firmness among different transgenic lines. The results revealed that the parenchyma cells of *FvePL1/4/7-*ox cortex were outstandingly more giant, obviously separated, and sparsely arranged. Conversely, smaller intercellular spaces and fewer contact areas between adjacent cells were noticed in *FvePL1/4/7*-*RNAi* transgenic fruits (Fig. 8b). The fruit firmness of RNAi was increased by 38% in average over the wild-type. By contrast, the fruit firmness of *FvePL1/4/7s-*ox fruit was substantially lower than WT, suggesting that *FvePL1*, *4*, and *7* negatively regulated fruit firmness (Fig. 8c). The increased cell division and expansion might contribute to reducing cell wall texture and fruit firmness.

### *FvePL1 *is related to the vegetative and pollen development of *F. vesca*

**Fig. 9:**
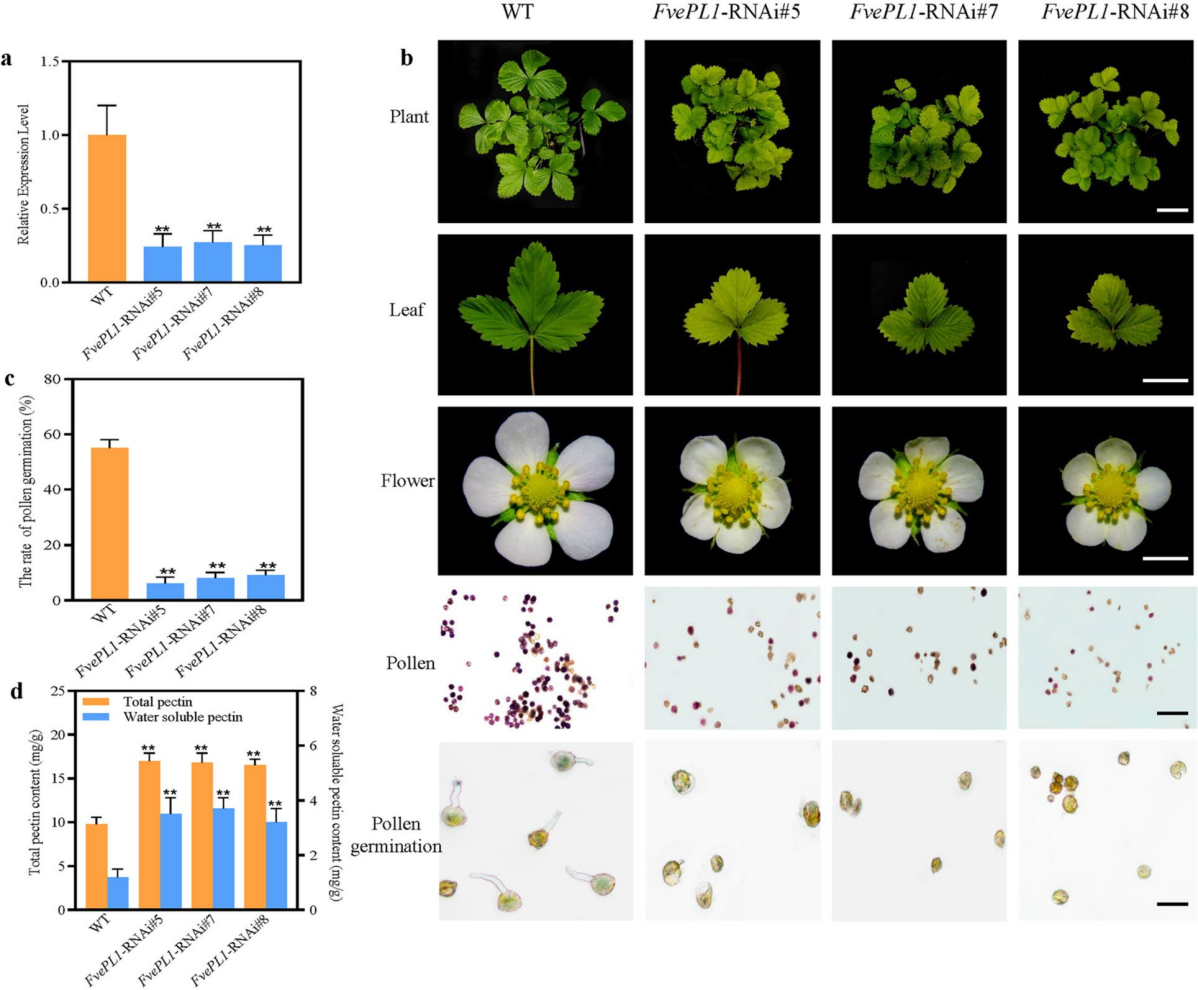
Phenotypic characterizations of the RNAi.** a** The relative expression levels of *FvePL1.* **b** Seedlings (bar = 5 cm), leaf (bar = 2 cm), open flowers (bar = 0.5 cm), I2-KI staining of pollen (bars = 100 μm) and pollen germination (bars = 200 μm). **c** The rate of pollen germination. **d** The content of total pectin and water-soluble pectin extracted from leaves of wild-type and RNAi lines

To genetically validate the functions of *FvePL*, *FvePL1-*RNAi was constructed and stably introduced in the YW background individually. *FvePL1* expression levels were detected by qRT-PCR (Fig. 9a). Compared with wild-type plants, *FvePL1* was significantly down-regulated in transgenic lines. The lowest *FvePL1* level was almost a four-fold decrease. During vegetative growth, RNAi displayed dwarf seedlings and smaller leaves (Fig. 9b). Petals became wrinkled, and fewer pollen grains were normally developed in *FvePL1-* RNAi lines. Some pollen grains could not be deeply stained with MTT and had exhibited irregular, shrunken shapes, resulting in a partial male sterile phenotype. By observing the gemination tube, the rate of pollen germination was calculated and substantially decreased by 89.09%, 85.45% and 83.64% in *FvePL1-*RNAi#5, #7, #8, respectively (Fig. 9c). These phenotypes suggested that *FvePL1* was involved in the development of seedlings and influenced pollen maturation. Finally, we measured the contents of total and water-soluble pectin extracted from leaves of different genotypes (Fig. 9d). Compared with the wild-type, the total pectin and water-soluble had averages of 41.14% and 65.42% increases in transgenic lines, suggesting that *FvePL1* might promote cell size as a result of increased cell expansion by degrading the pectin.

## Discussion

### Systematic and comprehensive genome-wide detection of FvePLs

In the current study, sixteen *PL* members were identified from the *F. vesca* genome. The number was fewer than cotton (83), Arabidopsis (26), poplar (30), and tomato (22), even though *F. vesca* genome size (~ 240 Mb) has a 64.38% increase compared with Arabidopsis (146 Mb). FvePLs were divided into four groups based on phylogenetic analysis. The similarities of the gene structures, domains, and motifs of *FvePLs* branched into the same group and contributed to conserved functions due to a long evolutionary history or gene duplication. *FvePL6* and *8* were excellent examples of this, given that they clustered in the same group and were all preferentially expressed in mature anther and pollen grains according to spatial-temporal expression and in situ hybridization analysis.

The syntenic maps revealed that no tandem duplication was observed for any pair of *FvePLs*, specifying that WGD or segmental duplication contributed to the expansion of *FvePLs*. Similar duplication events were also reported in the *PtPLs* development in Poplar [8]. The copies of *FaPLs* orthologous to *FvePLs* ranged from two to ten. Besides, the ratio of sequences with different chromosome numbers between *F. vesca* and *F. ananassa* varied from 1:1 to 1:3, thus suggesting that *F. vesca* may be one ancestor and the dominant sub-genome of cultivated strawberry octoploid *F. ananassa*. Recently the origin of cultivated strawberry was studied by high-quality, telomere-to-telomere, and gap-free *F. vesca* genome [37]. The results showed that allopolyploid *F. ananassa* originated from only two diploid ancestors, *F. vesca* and *F. viridis*, not the previous four ancestors [38]. Three sub-genomes of *F. ananassa* belong to the *F. vesca* group, and one is sister to *F. viridis*, corresponding to our conclusion that *F. vesca* is the closest ancestor of the cultivated strawberry. *FvePL6*, 8 and *13* contributed the most significant expansions of *PLs* comparing orthologous *PL* genes from *M. domestica*, *S. lycopersicum* and Arabidopsis. Interestingly, the co-expression network showed that the three highest links of *FvePLs* were *FvePL6*, *13* and *8* (correlated with 727, 707, and 655 genes, respectively), thus suggesting that *FvePL6*, *8* and *13* may be substantially crucial for the evolution of the *PL* gene family and play essential roles in strawberry development (Additional file 7). Considering that genomic comparisons with orthologous genes from model plant species may provide a valuable reference, we found several orthologous gene pairs of *F. vesca* between Arabidopsis or other species, indicating that *FvePLs* genes in question shared a common ancestor and conserved functions during evolutionary development [39]. For example, *SlPL9* (*Solyc03g111690*) was highly expressed during fruit maturation and negatively regulated fruit firmness [16]. Consistently, its orthologous gene *FvePL7* showed dominant expression in fruit at the turning stage and proved to accelerate fruit ripening by transgenic lines in this study. These approaches would be feasible to prioritize other *FvePLs* for further functional genomics studies of strawberries.

### *FvePLs *promoters were active with hormones to regulate strawberry development

The strawberry fruit is modified mainly by ABA from receptacle to achene, although ethylene and auxin are also implicated in the process [40]. ABA is a major ripening regulator in non-climacteric fruit on account of enhancing fruit ripening rapidly [41]. All *FvePLs* promoters contained at least one ABA-responsive element, inferring that their expression might be responsive to ABA metabolism, signaling, and transport. The most highlight was the promoter region of *FvePL4* harboring 15 ABA-responsive elements (ten MYB2CONSENSUSAT and five MYCATRD22). Correspondingly, the previous study proved that ABA signaling induced the transcripts of *FveWRKY48* that bound to the *FvePL4* promoter to degrade pectin [42].

*PLs* are generally induced by auxins, leading to cell wall loosening and organ initiation [43]. The promoter regions of the *FvePLs* in this study also contain multiple auxin-responsive elements. *FvePL1* promoters harbored seven auxin-responsive elements, including four CATATGGMSAUR, two RYREPEATVFLEB4, and one ARFAT (Fig. 3). Similarly, *ZePLs* were auxin-inducible to promote cell elongation and differentiation in the *Zinnia* mesophyll cell system [43]. Besides, all *GhPLs* significantly responded to IAA treatment to promote fiber elongation and anther development at the meiotic stage [44]. *FvePL8* promoter harbored abundant auxin-responsive elements (four CATATGGMSAUR and four ARFAT elements). It was exclusively expressed in pollen grains, suggesting that *FvePL8* may take part in anther dehiscence, cell wall loosening in pollen, pollen tube elongation and the promotion of pollen penetration through style tissue degradation. Additionally, the co-expression networks revealed that several *FvePLs* were closely correlated with pectinesterase and auxin-induced proteins (Additional file 7). Thus, hormones play an essential role in the manipulation of *FvePLs* expression to determine strawberry growth and development.

### Functional and regulatory divergence of *FvePLs *in anther development and fruit softening

Gene expression profiles can provide important clues to reveal potential gene functions. Transcripts of *FvePLs* genes in group I were relatively low in most tissues, indicating pseudo-functionalization [45]. Despite higher expressions of *FvePL2*, *3*, *9, 10* and *14* in some specific tissues compared to any other tissues, their absolute lower expressions suggest slight roles in the development of strawberry. However, the other three genes were exclusively expressed in specific organs. *FvePL6, 8* and *13* contributed to the significant expansions during evolution. They were highlighted due to their abundantly expressed in reproductive organs, in accordance with the functions of most *PLs* in *Brassica rapa* and Arabidopsis [46]. Their higher expressions might promote pollen germination by loosening the cell wall to allow pollen tube emergence and growth to facilitate penetration of pollens. Some *FvePLs* were reported to play critical roles in floral and vascular developments. *FvePLs* in group II belong to constitutive expressions, suggesting their important roles in the entire development of strawberry, especially pollen maturing and fruit ripening.

The remarkable expression of some *FvePLs* in fruit ripening also indicates their substantial roles in fruit development. Slowing fruit softening to extend shelf life remains a major challenge for strawberry improvement. A practical approach to control strawberry softening was manipulating *FvePL* genes [12]. The reduction of the steady-state levels of *FvePL1, 4* and *7* resulted in a high increase in firmness and reduced postharvest softening. However, *FvePLs* negatively regulate fruit firmness might through a different molecular mechanism. For example, ABA-induced protein FvWRKY48 binds to the *FvPL4* promoter via a W-box element to control fruit softening and pectin degradation in *F. vesca*, but this is probably not the exact mechanism of *FvePL1* or 7 due to the connection with different transcriptional factors [47]. Finally, the data suggested that alternative mechanisms might regulate *FvePLs* expression to modulate fruit ripening, though experimental data were still necessary to assess the predictions.

### Supplementary Information

**Additional file 1.**

**Additional file 2.**

**Additional file 3.**

**Additional file 4.**

**Additional file 5.**

**Additional file 6.**

**Additional file 7.**

## Data Availability

The protein sequences of *F. vesca* (v4.0.a2) were downloaded from the Genome Database for Rosaceae (GDR) website at www.rosaceae.org/species/fragaria/fragaria_vesca. Illumina reads of all samples have been submitted to the Sequence Read Archive at NCBI (http://www.ncbi.nlm.nih.gov/sra). The accession numbers are SRA065786, SRP035308, and SRR5155708 to SRR5155715.
